# Illuminating the dark majority: photobiology of nonphotosynthetic bacteria

**DOI:** 10.1007/s43630-025-00789-6

**Published:** 2025-10-20

**Authors:** Edoardo Cianflone, Giuseppe Maria Paternò

**Affiliations:** 1 Department of Physics, Politecnico di Milano, Piazza Leonardo Da Vinci 32, 20133 Milan, Italy; 2 Center for Nanoscience and Technology, Istituto Italiano di Tecnologia, Via Rubattino 81, 20133 Milan, Italy

## Abstract

Nonphotosynthetic bacteria represent the majority of prokaryotic diversity across virtually all terrestrial and host-associated environments, yet their interactions with light have long been overlooked. Recent discoveries have revealed that, even in the absence of canonical photosystems, these organisms possess an array of photoreceptors that couple light sensing to rapid membrane-potential changes, stress-response pathways, gene regulation, and motility. Here, we review three principal classes of light-induced phenomena in nonphotosynthetic bacteria: (1) electrophysiological responses, where light stimulation elicits membrane-potential modulation; (2) stress-response modulation, including light-activated sigma factors, DNA-repair enzymes, antioxidant defences and circadian-like and anticipatory transcriptional programs that align physiology with environmental light–dark cycles; (3) phototaxis, in which light gradients rewire chemoreceptor signaling to bias flagellar rotation. By illuminating how nonphotosynthetic bacteria exploit light as an environmental cue, we aim to inspire new strategies in antimicrobial therapy, synthetic biology, and environmental biotechnology.

## Introduction

Nonphotosynthetic bacteria constitute the vast majority of prokaryotic life on Earth, dominating virtually every habitat, from the dark depths of the subsurface to human-associated niches, except for sunlit ocean surfaces, where photosynthetic microbes prevail [1–3]. Model organisms such as *Bacillus subtilis*, *Escherichia coli* and *Pseudomonas aeruginosa*, along with many of our most troublesome pathogens (e.g. *Clostridioides difficile*, *Vibrio cholerae*), fall into this nonphotosynthetic majority. Normally, their interactions with light were thought to be negligible, since they do not harness photons for growth, and therefore often went unexamined.

Conversely, a growing body of evidence is revealing that even “nonphotosynthetic” bacteria possess light-sensitive, excitable components in their cell envelopes. These findings opened a new avenue of research into how light can modulate bacterial physiology and behavior, despite not serving as an energy source. It is important saying that this intrinsic light sensitivity of nonphotosynthetic bacteria electrophysiology must be carefully considered when introducing additional light-responsive elements, such as exogenous photoreceptive interfaces [4–12], since it may interact with or confound engineered optoelectronic modulation strategies.

**Fig. 1:**
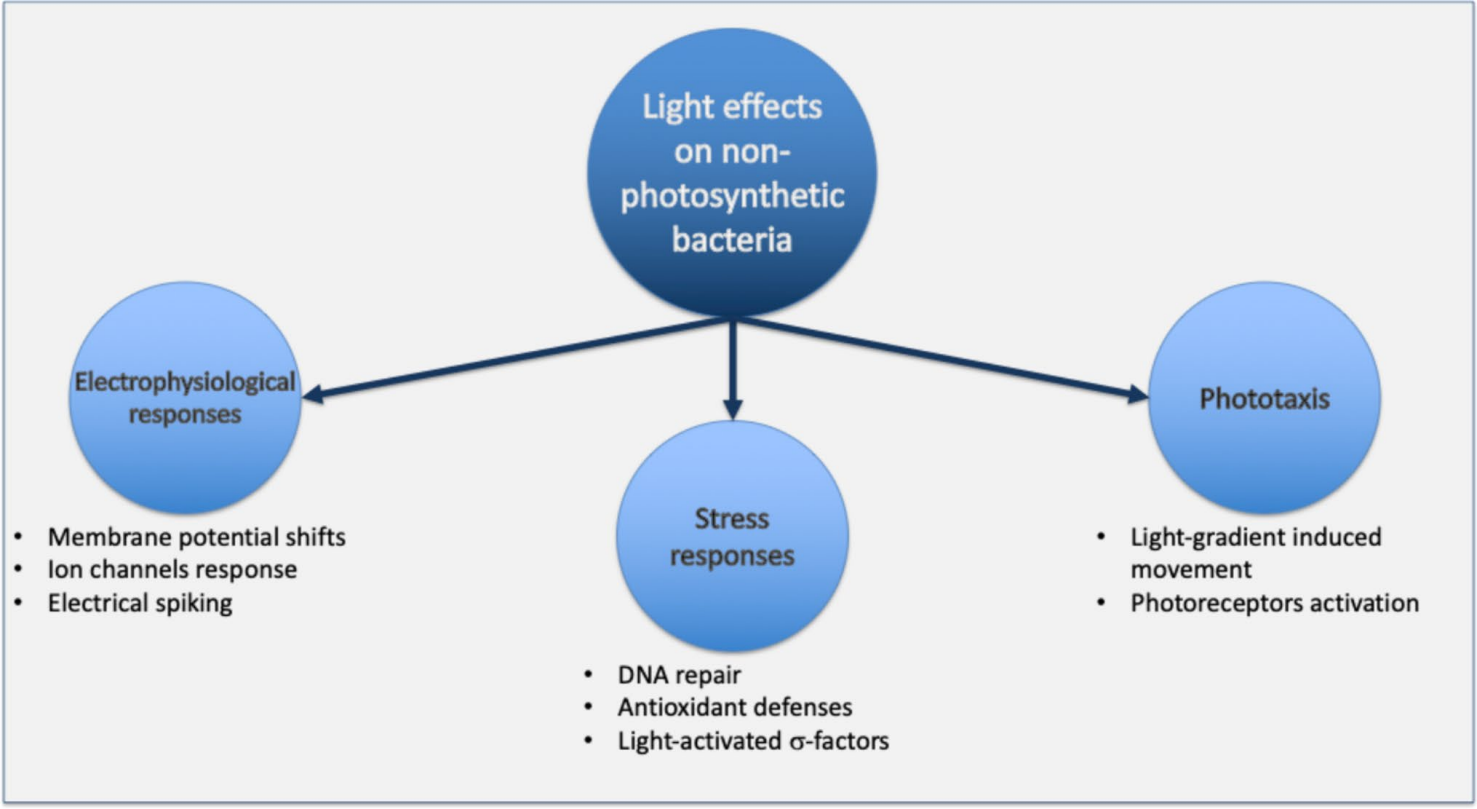
Schematic overview of light effects on nonphotosynthetic bacteria. Light exposure influences three major functional domains: electrophysiological responses (membrane-potential shifts, ion channel activity, and electrical spiking), stress responses (DNA repair, antioxidant defenses, and activation of stress σ-factors), and phototaxis (light-gradient–dependent motility mediated by photoreceptor activation). Together, these processes highlight light as a key environmental cue shaping bacterial physiology and behavior

In this review, we explore three major classes of light-induced phenomena in nonphotosynthetic bacteria (Fig. 1):

- *Electrophysiological responses*—how membrane potential and ion channels respond to specific wavelengths.
- *Stress-response modulation and regulation*—including DNA repair pathways and oxidative defences activated by ultraviolet and visible light.
- *Phototaxis*—directional movement in response to light gradients.

Because nonphotosynthetic bacteria employ diverse metabolic strategies, their light responses are embedded within varied ecological and physiological contexts. Broadly, they can be grouped as follows (see Table 1).

**Table 1:** Overview of nonphotosynthetic bacterial metabolic classes: energy sources, model taxa, and ecological niches

Metabolic class	Energy source	Representative taxa	Typical habitats
Chemoheterotrophs [13]	Oxidation of organic compounds	*E. coli*, *Staphylococcus aureus*	Soil, gut, marine detritus
Chemoautotrophs [14]	Oxidation of inorganic substrates	Nitrifiers, sulfur oxidizers	Deep-sea vents, sediments
Nitrate-reducing bacteria [15]	Nitrate → nitrite	*Paracoccus denitrificans*, diverse Proteobacteria	Aquatic, wastewater systems
Sulfate-reducing bacteria [16]	Sulfate → H₂S	*Desulfovibrio* spp.	Anoxic sediments, gut
Iron-reducing bacteria [17]	Fe^3^⁺ → Fe^2^⁺	*Geobacter* spp., *Shewanella* spp.	Sediments, subsurface aquifers

Despite the ubiquity of these strains in laboratories and clinics, their “nonphotosynthetic” label has often obscured the light-sensitive facets of their biology. In what follows, we synthesize recent advances that reveal how, even without canonical photosystems, these organisms deploy, and in many cases exploit, light as a versatile environmental cue.

Emerging evidence now positions light not just as an irrelevant environmental background, but as a fundamental signal shaping the physiology, behavior, and ecology of nonphotosynthetic bacteria. By integrating electrophysiological measurements, genetic dissection of photoreceptors, and ecological studies in diverse habitats, we are beginning to uncover how light influences nutrient cycling, microbial community dynamics, and host–microbe interactions. Ultimately, understanding these light-mediated processes may yield novel strategies for antimicrobial interventions, light-based tools in microbiology, and bioengineering applications that harness bacterial light responses. We anticipate that, as this field matures, light will join the ranks of classical stimuli, such as nutrients and signaling molecules, as a key driver of bacterial life.

## Light dependence of electrophysiological responses

The recent studies have revealed that nonphotosynthetic bacteria exhibit rapid, light-dependent changes in membrane potential, akin to the action potentials of neurons [18, 19]. Kralj et al. (2011) [20] pioneered this field by using a genetically encoded voltage-sensitive fluorescent protein (VSFP) to show that electrical stimuli pulses induced membrane potential modulation in *Escherichia coli*, implicating excitability of bacteria. Building on this, Prindle et al. (2015) [21] demonstrated that coordinated oscillations of membrane potential can propagate across *Bacillus subtilis* biofilms, suggesting a mechanism for long-range electrical signaling in microbial communities.

**Fig. 2:**
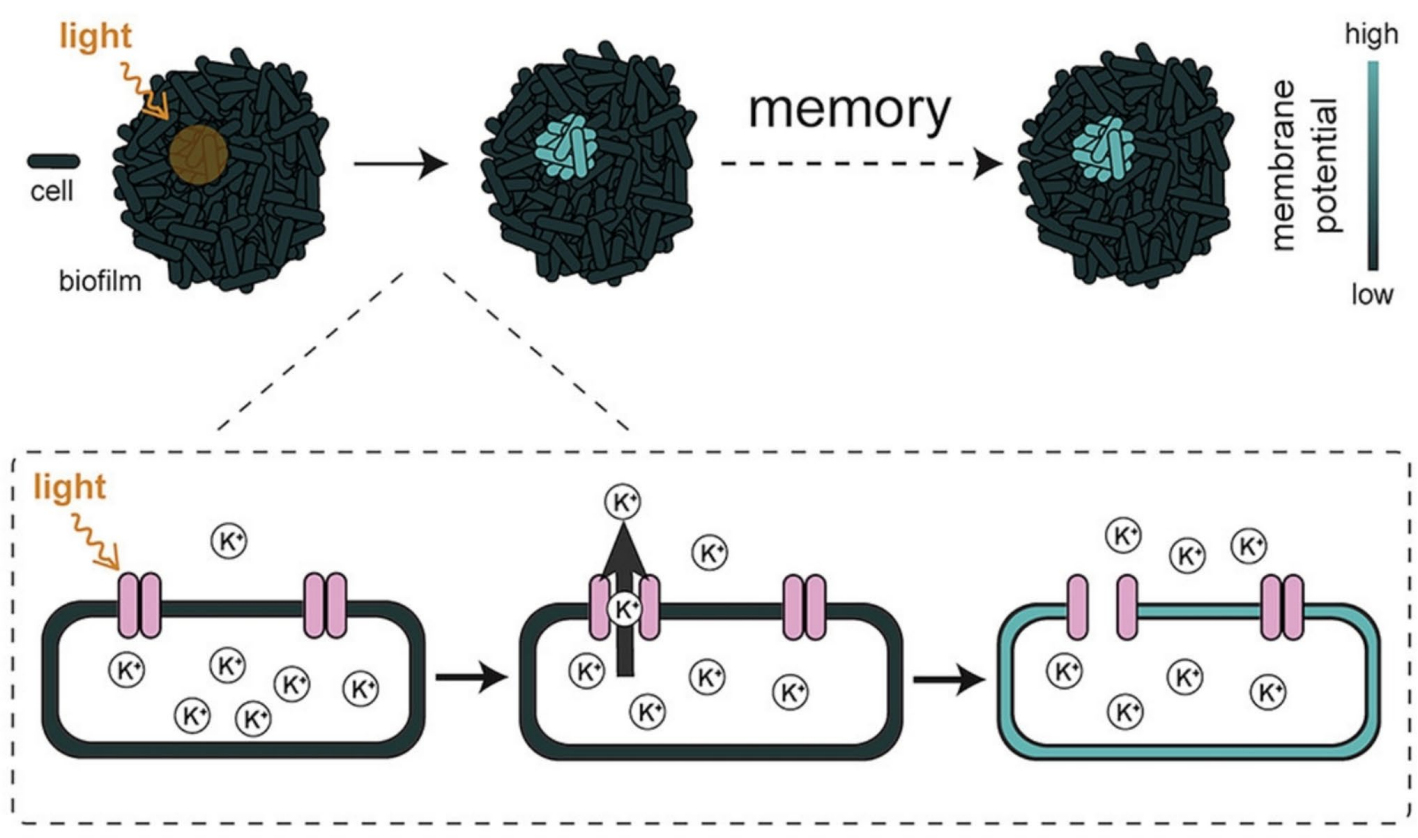
Yang et al. revealed that brief light pulses can “write” single-cell memory patterns into bacterial biofilms by shifting their membrane potential. Building on this, they showed that a momentary optical stimulus elicits a durable, potassium-channel–driven hyperpolarization that persists for hours and can be visualized at single-cell resolution as a stable spatial pattern. Such long-lived, membrane-potential-encoded memories hint at the possibility of distributed computations within prokaryotic communities, drawing an intriguing parallel between neural networks and bacterial biofilms. Reproduced from Ref. under permission of Elsevier (2020) [22]

Blue-light exposure triggers profound changes in bacterial membrane potential across multiple species [22]. In *Bacillus subtilis*, this causes membrane hyperpolarization within seconds through increased potassium efflux via the YugO potassium channel (Fig. 2) [10, 23]. This hyperpolarization is specific to blue-light wavelengths (~438–480 nm) and increases with light intensity. Remarkably, this behavior persists for hours after a brief optical stimulus, allowing for single-cell resolution mapping of spatial memory patterns within the biofilm. The capacity to encode stable and long-lasting membrane potential–based memory suggests that bacterial communities may be capable of performing distributed computations, drawing a striking parallel between bacterial biofilms and neural networks. The authors attribute this response to the LOV (Light, Oxygen, Voltage) domain-containing protein YtvA, which activates the σ^B^-controlled stress-response pathway.

Similarly, *Escherichia coli* exhibits light-induced membrane-potential oscillations and spiking behavior when exposed to blue light. These dynamics are characterized by initial hyperpolarization followed by depolarization, with the patterns being mediated by the voltage-gated Kch potassium channel [24]. In another study, Blee et al. found that exposure to 405 nm light triggered both membrane hyperpolarization and cell dispersal in *Pseudomonas aeruginosa* and *Bacillus subtilis* biofilms [25]. These responses were dependent on the biofilm’s developmental stage, suggesting a dynamic sensitivity to light over time. Notably, the application of reactive oxygen species (ROS) scavengers attenuated both the hyperpolarization and the dispersal effects, indicating that ROS play a key role in mediating the electrophysiological response to 405 nm light. In-fact a stress-response mechanism to blue-light seems to be a general paradigm in nonphotosynthetic bacteria, as this was firstly observed by Ávila-Pérez et al. in 2006 [26]. A more detailed overview on the light-induced stress mechanisms in nonphotosynthetic bacteria is presented in the next section.

## Light-dependent stress-response

Though light is not essential for energy generation in nonphotosynthetic bacteria, many of these organisms possess sophisticated light-sensing systems to respond to its presence [27]. Exposure to light, can trigger oxidative stress, damage cellular components, and regulate circadian clocks. To deal with such conditions, nonphotosynthetic bacteria have evolved photoreceptors, stress-response sigma factors, DNA repair systems, and light-regulated gene expression networks. These responses often parallel phototrophic systems in terms of protective mechanisms, despite the absence of photosynthesis. The most relevant mechanisms of stress-response are summarized in Table 2.

**Table 2:** Key mechanisms in light stress response of nonphotosynthetic bacteria

Mechanism	Role in light stress response
Photoreceptors (BLUF, LOV, Phytochromes)	Sense light and activate downstream transcriptional regulators
Sigma Factors (RpoH, SigB)	Drive expression of stress-response genes including chaperones and antioxidants
DNA Repair Systems (Photolyases, UvrA/B)	Repair UV-induced DNA damage
Antioxidant Systems (SOD, Catalase, Dps)	Detoxify ROS produced by light exposure
Circadian Regulation	Align physiological activities with environmental light–dark cycles
Carotenogenesis	Protects cellular membranes by quenching ROS

The seminal review by van der Horst, Key, and Hellingwerf (2007) expanded our understanding of bacterial sensory biology by demonstrating that photosensing is not exclusive to photosynthetic organisms. The discovery that chemotrophic bacteria possess sophisticated light-sensing systems across all major photoreceptor families suggests that temporal and spatial light information represents a universal environmental signal in bacterial ecology. Most bacterial photoreceptor proteins are modular, combining light-sensing input domains (such as BLUF, GAF, EAL, and GGDEF) with enzymatic or regulatory output domains, enabling diverse light-mediated signaling functions. The modular architecture of bacterial photoreceptors, coupled with their diverse physiological functions ranging from stress responses to virulence regulation, highlights the evolutionary significance of light sensing in bacterial adaptation [28].

Sigma factors RpoH and SigB are critical transcriptional regulators that enable nonphotosynthetic bacteria to exhibit adaptive responses to environmental stress, including light-induced oxidative damage. RpoH (σ^32) is well known for controlling the heat shock response by upregulating chaperones and proteases that maintain protein homeostasis under stress [29]. In *Azospirillum brasilense*, a soil-dwelling nonphotosynthetic rhizobacterium, the alternative sigma factor RpoH2 regulates photooxidative stress responses, including activation of genes encoding antioxidant defence enzymes such as catalase and superoxide dismutase [30]. SigB (σ^B), a general stress sigma factor in Gram-positive bacteria like *B. subtilis*, is activated by a range of stimuli including light, salt, oxidative, and energy stress [31]. Upon activation, SigB mediates transcriptional reprogramming by guiding RNA polymerase to stress-responsive promoters, inducing genes responsible for detoxifying reactive oxygen species, maintaining redox balance, and stabilizing macromolecules [31]. Notably, SigB activity has been linked to light sensing through interactions with blue-light photoreceptors like YtvA, connecting environmental light cues to general stress regulation [26]. Together, RpoH and SigB exemplify the versatile and modular strategies bacteria employ to deal with abiotic stress, even in species lacking photosynthetic machinery.

DNA repair systems such as photolyases and the UvrABC endonuclease complex are essential defence mechanisms in nonphotosynthetic bacteria exposed to UV and visible light. Ultraviolet (UV) radiation can induce DNA lesions like cyclobutane pyrimidine dimers (CPDs) and 6-4 photoproducts, which if unrepaired, lead to mutations or cell death. Photolyases, a class of flavoproteins, directly reverse CPDs using energy derived from blue light in a process known as photoreactivation [32]. These enzymes are widespread across bacterial taxa, including many nonphototrophic soil and aquatic microbes. In contrast, UvrA, UvrB, and UvrC form the core of the nucleotide excision repair (NER) pathway, which operates independently of light by recognizing and excising bulky DNA lesions, followed by DNA synthesis and ligation [33]. UvrA acts as the damage sensor, UvrB verifies and unwinds DNA, while UvrC performs dual incisions around the lesion [34]. Notably, these systems often work in tandem: photolyases address specific UV-induced lesions under light, while UvrABC provides broader damage repair in both light and dark conditions. The conservation and inducibility of these pathways highlight the evolutionary importance of genomic integrity in nonphotosynthetic bacteria inhabiting light-variable environments [35].

Antioxidant systems in nonphotosynthetic bacteria, including superoxide dismutase (SOD) and catalase are central to mitigating light-induced oxidative stress. Reactive oxygen species (ROS), such as superoxide anions and hydrogen peroxide, are common byproducts of UV or visible light exposure, even in bacteria that do not rely on photosynthesis. SOD enzymes convert superoxide radicals into hydrogen peroxide, which is then detoxified by catalase or peroxidases into water and oxygen, preventing cellular damage. For instance, *Rhodococcus erythropolis*, a nonphotosynthetic actinobacterium, upregulates SOD and catalase activity when exposed to light, enhancing survival under photooxidative conditions [36]. These defence mechanisms are often transcriptionally regulated by light-sensing proteins and stress-response sigma factors, enabling anticipatory or responsive regulation depending on environmental cues [37]. The high conservation and inducibility of these systems across nonphototrophic taxa underline their evolutionary importance in oxidative stress resilience.

**Fig. 3:**
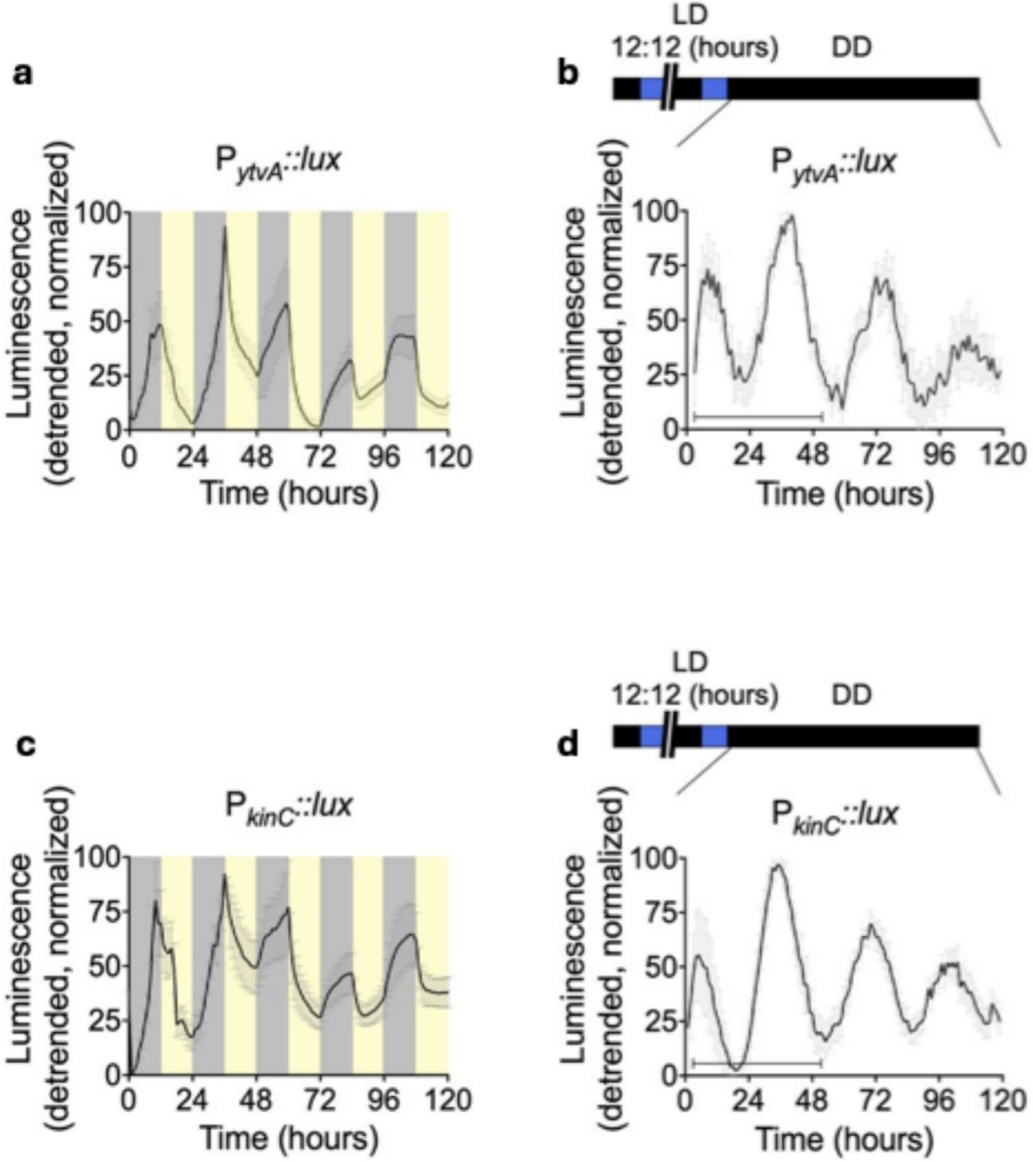
Bioluminescence rhythms of *PytvA::lux* (**A–C**) and *PkinC::lux* (**D–F**) during a 5-day entrainment to 12 h dark/12 h blue-light cycles (**A, D**) followed by 5 days in constant darkness (DD; **B, E**). All assays were conducted at a constant 25.5 °C. Data are detrended and plotted as mean ± SD. In panels A and D, yellow and gray shading denote light and dark phases, respectively. In panels B and E, the black bar (lower left) indicates the 48-h interval used to calculate the free-running period. Reproduced from Ref. under Creative Commons License [38]

Eelderink-Chen et al. (2021) provided the first clear demonstration of a circadian clock in a nonphotosynthetic bacterium, *B. subtilis* (Fig. 3) [38]. Their study showed that this bacterium exhibits all canonical properties of circadian systems: free-running rhythms, entrainment to light and temperature cycles, and temperature compensation. Using luciferase reporter strains, the authors revealed that gene expression in these bacteria can be synchronized to 24-h environmental cycles and that, upon release into constant conditions, biofilm populations display robust, temperature-compensated oscillations with periods close to 24 h. This discovery not only challenges the assumption that circadian clocks are absent in nonphotosynthetic prokaryotes but also opens new avenues for understanding temporal regulation in bacterial physiology, with potential implications for biomedicine, ecology, and industrial processes.

More recently, Hatfield et al. (2023) demonstrated that nonphotosynthetic terrestrial bacteria, including *Pseudomonas syringae*, can use light as an anticipatory cue to prepare for impending environmental water loss. The authors found that exposure to various light wavelengths triggers broad transcriptional reprogramming, largely mediated by a bacteriophytochrome photoreceptor, resulting in the upregulation of water-stress adaptation pathways before desiccation occurs. This proactive light-sensing strategy enhances bacterial fitness by allowing cells to pre-emptively adapt to predictable, ecologically coupled stresses such as rapid post-dawn evaporation, highlighting an advanced form of environmentally driven anticipation in nonphotosynthetic microbes [39].

Carotenogenesis in nonphotosynthetic bacteria is a protective metabolic strategy that mitigates light-induced oxidative stress by producing carotenoid pigments with strong antioxidant properties. While traditionally associated with phototrophic organisms, carotenoid biosynthesis is increasingly recognized in nonphotosynthetic bacteria such as *Myxococcus xanthus*, *Streptomyces coelicolor*, and *Deinococcus radiodurans* [40, 41]. In these microbes, exposure to UV or visible light can lead to the generation of reactive oxygen species (ROS), which carotenoids neutralize by quenching singlet oxygen and lipid radicals. Recent studies have revealed that light acts as a direct trigger for the upregulation of carotenoid biosynthetic genes through blue-light photoreceptors and regulatory proteins such as CarH or Blue Light Using FAD (BLUF) domains [42]. In *M. xanthus*, for instance, light activates a transcriptional cascade that coordinates carotenoid synthesis in response to singlet oxygen stress, with protoporphyrin IX acting as an internal photosensitizer [43]. These findings emphasize that, even in the absence of photosynthesis, carotenogenesis serves a crucial photoprotective role, enabling bacteria to survive fluctuating light environments, especially in surface soils or exposed niches.

## Phototaxis in nonphotosynthetic bacteria

**Fig. 4:**
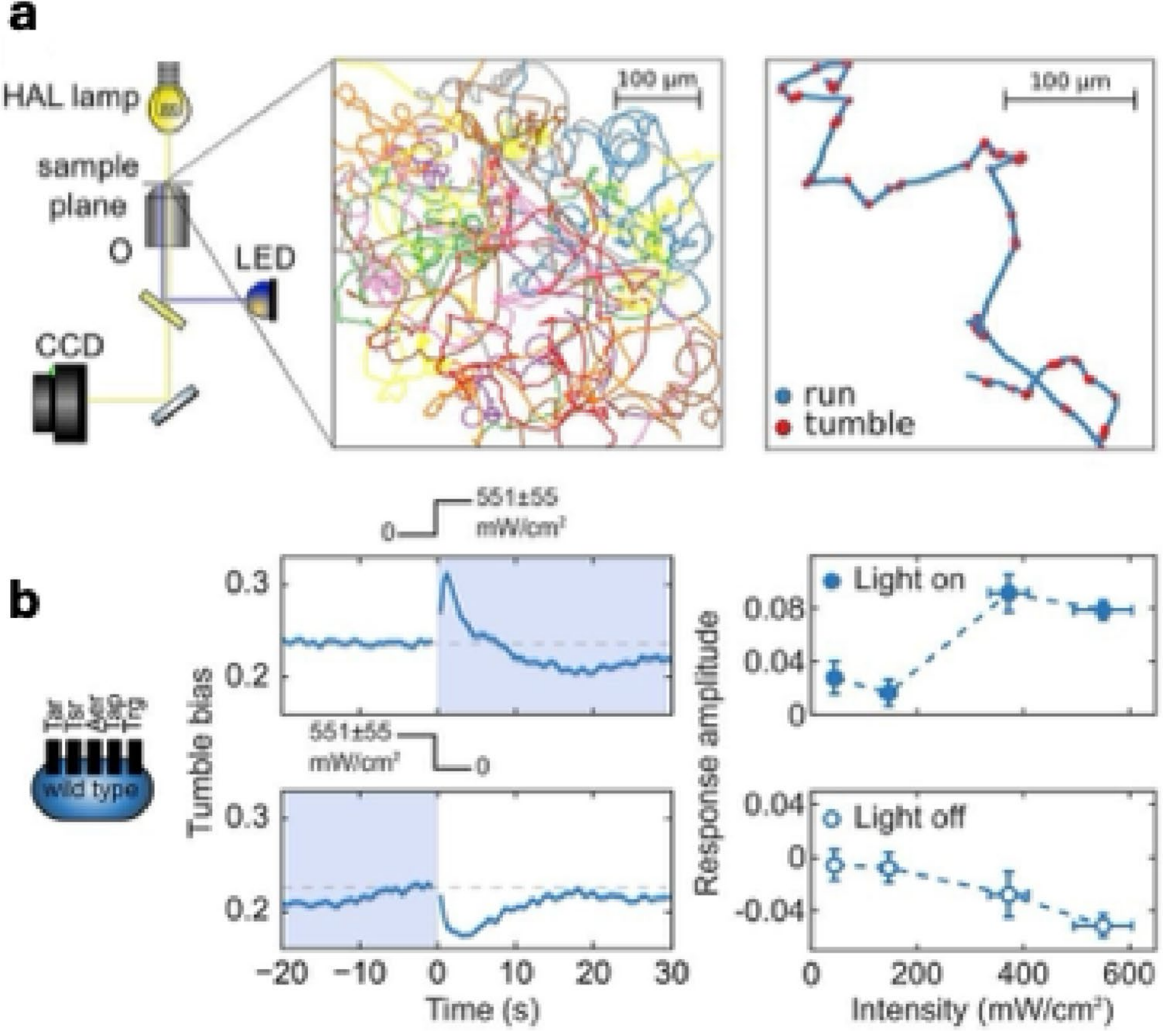
Phototaxis in *E. coli*. **A** Experimental setup and data analysis for investigating phototaxis in *E. coli*. A wide-range halogen (HAL) lamp (yellow light path) and a blue LED (blue-light path) are integrated into an inverted light microscope equipped with a 20× objective (O) to simultaneously image and stimulate swimming cells. Bacterial motion is recorded using a CCD camera. Individual cells are detected in each video frame, and their positions are linked to reconstruct trajectories, which are then filtered and analyzed to classify runs and tumbles. **B**, left and centre: Phototactic response of the wild-type *E. coli* strain RP437 to blue light (551 ± 55 mW/cm^2^), showing behavioral changes upon light onset and offset. Shaded regions and intensity traces above the plots indicate periods of illumination. Each point represents the average tumble bias of ~6000–7000 trajectories, with shaded areas denoting the standard error of the mean. **B**, right: Quantification of response amplitude to light onset (top) and offset (bottom) across a range of light intensities. Adapted from Ref. under the permission of the American Society of Bacteriology (2019) [45]

Although nonphotosynthetic bacteria lack photosystems, many have evolved sophisticated ways to interpret light as a navigational cue and adjust their motility accordingly. In *E. coli*, blue light (≈ 390–530 nm) perturbs the proton-motive force and is therefore “felt” by all five chemoreceptors; the net output of their competing signals biases flagellar rotation so that populations tumble or run and rapidly redistribute within a gradient, making blue light a truly universal phototactic stimulus for this species (Fig. 4) [44, 45].

Yang et al. (1995) demonstrated that *E. coli* mutants defective in ferrochelatase (hemH) exhibit negative phototaxis away from blue light due to accumulated protoporphyrin IX [46]. These mutants showed rapid tumbling responses within 1 s of blue-light exposure (396–450 nm) and subsequent running behavior upon light removal. The wavelength specificity matched the absorption spectrum of protoporphyrin IX, which accumulates to over 100-fold higher levels than wild-type cells. This illustrates how endogenous photosensitizers can amplify avoidance behavior. Other heterotrophs display comparable strategies: *Serratia marcescens* swarms retreat from high-intensity visible light by modulating collective flows, while many purple bacteria and haloarchaea combine photophobic and scotophobic turns to settle in niches where illumination is neither too dim nor DNA-damaging [47].

*B. subtilis* adds an intriguing Gram-positive perspective. Here the blue-light sensor YtvA, a LOV–STAS protein, feeds into the σ^B stressosome; even brief exposure elevates σ^B activity, down-regulates motility genes, and reduces swimming speed, effectively discouraging cells from remaining in brightly lit microenvironments [26]. Under stronger or longer illumination, a complementary RsbP-dependent pathway responsive to red light further broadens the spectral range that can suppress motility, hinting at layered photo-avoidance circuits adapted to the soil’s diffuse light field [48, 49].

Wood et al. (2018) demonstrated that *Acinetobacter baumannii* senses and responds to light via a blue-light BLUF photoreceptor, BlsA, which regulates expression of the photoregulated pilus operon (prpABCD) [50]. This light-sensitive operon is critical for type I pilus assembly, influencing surface motility, pellicle formation, and biofilm development. Their study showed that in darkness at 24 °C, prpABCD expression is upregulated, promoting motility and pellicle formation while limiting biofilm production on plastic. Inactivation of prpA abolished light-regulated motility and reduced virulence in infection models. The findings reveal that *A. baumannii* leverages light-sensing through both BlsA-dependent and independent mechanisms to modulate surface interactions, biofilm behavior, and pathogenicity, supporting its adaptation to diverse ecological niches.

Together, these studies reveal that nonphotosynthetic bacteria exploit conserved chemotaxis modules, stress regulons or both to translate photon input into rapid electrical or transcriptional changes that steer individual cells, and sometimes entire communities, toward safer, metabolically favorable light regimes.

## Conclusions

In conclusion, it is now clear that nonphotosynthetic bacteria are intrinsically photosensitive: they possess a wide array of photoreceptors (BLUF, LOV, bacteriophytochromes) that link light detection to downstream effectors such as ion channels, stress sigma factors, DNA-repair enzymes and transcriptional regulators. For instance, blue-light stimulation can elicit rapid membrane‐potential shifts, hyperpolarizations, oscillations and spiking. At the same time, exposure to ultraviolet and visible light activates protective stress responses, including σ^B- and RpoH-mediated transcriptional programs, photolyase- and UvrABC-driven DNA repair, and antioxidant defenses based on superoxide dismutase, catalase and carotenoids. Light gradients also steer bacterial motility via chemoreceptor networks and stressosome pathways, guiding cells toward favorable microenvironments, while circadian-like rhythms in *B. subtilis* and anticipatory gene expression in surface‐dwelling microbes demonstrate that these organisms can both synchronize to and predict diel changes.

These discoveries carry important implications for synthetic and applied microbiology. Any attempt to interface bacteria with exogenous light-responsive elements, whether for optogenetic control, bioelectronic devices or light-based antimicrobials [51–61], must account for the native light sensitivity of bacterial electrophysiology and gene regulation to avoid unintended cross-talk or to exploit these responses for novel functionality. Regardless, there are still some limitations to face which are either practical, such as the limited dynamic range or the signal instability, or biological, for instance the phototoxicity or the variable response across species. Concerning the use of these systems as therapeutics, the scalability at an industrial level may be an issue to consider as well as the high variability in terms of light delivery and population-level responses that may be difficult to set outside of a laboratory environment.

Looking ahead, mechanistic studies of bacterial photoreceptors and ion channels, the development of tools for single-cell electrophysiology in situ, ecological investigations of light’s role in natural communities, and the translation of bacterial photobiology into programmable living materials and precision therapeutics all represent promising paths for future research. By recognizing light as an active environmental signal rather than an inert backdrop, we open new avenues for understanding the remarkable versatility of bacterial life.

## Data Availability

No datasets were generated or analyzed during the current study.
